# Fatal Measles Inclusion-Body Encephalitis in Adult with Untreated AIDS, France 

**DOI:** 10.3201/eid2609.200366

**Published:** 2020-09

**Authors:** Christophe Rodriguez, Meriadeg Ar Gouilh, Nicolas Weiss, Sébastian Stroer, Karima Mokhtari, Danielle Seilhean, Bertrand Mathon, Vanessa Demontant, Melissa N’Debi, Guillaume Gricourt, Paul-Louis Woerther, Jean-Michel Pawlotsky, Karl Stefic, Julien Marlet, Pierre-François Dequin, Antoine Guillon, Valérie Pourcher, David Boutolleau, Astrid Vabret, Sonia Burrel

**Affiliations:** Henri Mondor Hospital, Assistance Publique des Hôpitaux de Paris, University of Paris-Est, Créteil, France (C. Rodriguez, V. Demontant, M. N’Debi, G. Gricourt, P.-L. Woerther, J.-M. Pawlotsky);; National Reference Laboratory for Measles, Mumps, and Rubella, University Hospital of Caen, Normandie Université, Caen, France (M. Ar Gouilh, A. Vabret);; Pitié-Salpêtrière Hospital, Assistance Publique des Hôpitaux de Paris, Sorbonne-Université, Paris, France (N. Weiss, S. Stroer, K. Mokhtari, D. Seilhean, B. Mathon, V. Pourcher, D. Boutolleau, S. Burrel);; National Reference Center for Herpesviruses, Paris (D. Boutolleau, S. Burrel);; University Hospital of Tours, University of Tours, Tours, France (K. Stefic, J. Marlet, P.-F. Dequin, A. Guillon);; National Reference Center for HIV, Tours (K. Stefic, J. Marlet)

**Keywords:** measles inclusion-body encephalitis, MIBE, HIV/AIDS and other retroviruses, meningitis/encephalitis, viruses, brain biopsy, metagenomic, France

## Abstract

We report a fatal case of measles inclusion-body encephalitis occurring in a woman from Romania with AIDS. After an extensive but unsuccessful diagnostic evaluation, a pan-pathogen shotgun metagenomic approach revealed a measles virus infection. We identified no mutations previously associated with neurovirulence.

We report a fatal case of measles inclusion-body encephalitis in a 28-year-old woman from Romania living in France who had untreated AIDS. She was initially admitted to the hospital on September 22, 2018 (day 0), for afebrile generalized motor seizure that began focally in the right lower limb. Results of magnetic resonance imaging (MRI) were initially unremarkable, but electroencephalogram (EEG) results showed slight abnormalities related to slow frontal activity. The patient recovered fully and was discharged with antiepileptic therapy. Of note, the patient had stopped antiretroviral therapy (ART) 1 year earlier and declined to restart therapy after this hospital admission. 

In the next week, she had several relapses of focal seizures, requiring hospital readmission on day 7. Despite antiepileptic therapy adjustments, the myoclonic seizures persisted and became resistant to high doses of anticonvulsants and clonazepam add-on therapy (day 31). Consequently, the patient was hospitalized in intensive care unit. EEG results showed a pattern of frontal-lobe epilepsy. MRI results showed hyperintense cortical signals in frontal and left temporal cortex without hemorrhage lesions and without any signs of cerebral venous thrombosis. Biologic investigation revealed HIV replication and 26/mm^3^ CD4 T-cell count at day 35. We initiated antiretroviral medications on day 43. 

During her hospitalization, the patient showed a gradual impairment of consciousness (Glasgow coma score 6 on day 61) and was mechanically ventilated. MRI results showed increase of the cortical hyperintensities, and EEG results showed diffuse encephalopathy pattern (Figure 1). We analyzed cerebrospinal fluid (CSF) samples taken on days 37, 62, and 64 for pathogens: viruses (herpes simplex virus, varicella zoster virus, enterovirus, cytomegalovirus, Epstein-Barr virus, human herpesvirus 6, HIV, and polyomavirus JC), bacterial and mycobacteria, fungi (*Aspergillus* spp., *Cryptococcus neoformans*), and parasites (*Toxoplasma gondii*); no pathogens were detected. All CSF were paucicellular with protein and glucose levels within reference levels. Results of testing for autoimmune antibodies were also negative. 

**Figure 1:**
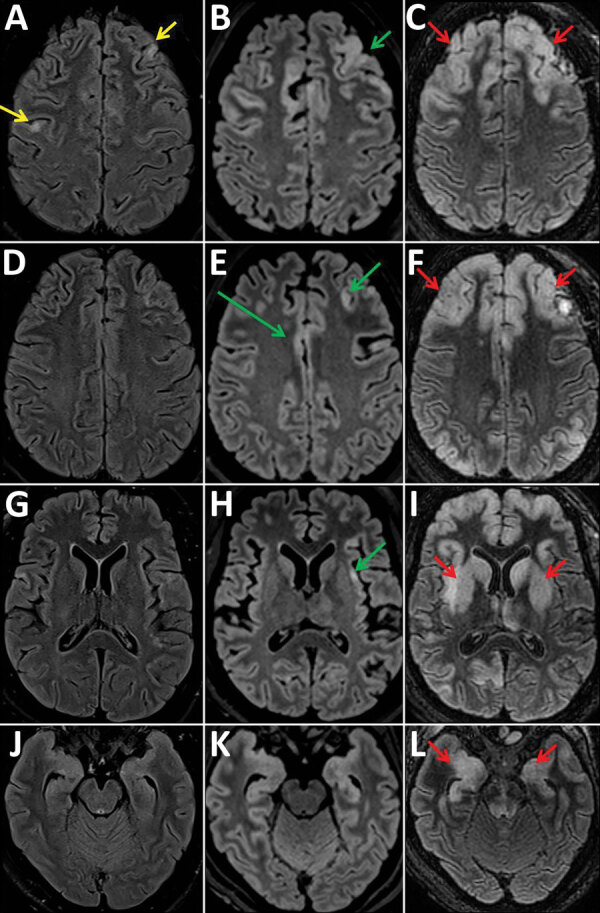
Fluid-attenuated inversion recovery images of magnetic resonance examinations of the brain in a 28-year-old woman from Romania with untreated AIDS and measles inclusion-body encephalitis. Images were taken 1 week (A, D, G, J), 2 weeks (B, E, H, K), and 5 weeks (C, F, I, L) after hospital admission, at the same brain levels. The first examination shows focal cortical hyperintensities (yellow arrows) in the left and right frontal cortex. After 2 weeks, these cortical hyperintensities have widened and are spreading to the cingulum and the insula (green arrows). At 5 weeks, cortical hyperintensities involve a larger part of the neocortex, but also spread to the basal ganglia, amygdala (red arrows), and hippocampus (the hippocampus changes may also be induced by status epilepticus) and to the posterior areas of the pons.

We performed a brain biopsy of the left frontal lobe on day 71 to determine the cause of encephalitis by the underlying neurologic symptoms, abnormal imaging features, and biologic findings. Neuropathology analysis revealed scarce inflammatory activation of glial cells (Figure 2). Because all the first-line microbiology testing assays remained negative on the biopsy, we considered using shotgun metagenomic (SMg) for panpathogen RNA/DNA detection to analyze the clinical samples with an unbiased approach. In brief, we performed an extraction combining bead beating and chemical and enzymatic lysis before library preparation. We performed sequencing on NextSeq500 with High Output Kit version 2.5 (300 cycles) (Illumina, https://www.illumina.com) (*1*). We analyzed sequencing data using MetaMIC software, which performed microorganism identification, genome reconstruction, and variant calling (*1*). A total of 5 samples were tested by SMg: CSF (day 61), bronchoalveolar lavage (days 63 and 76), brain biopsy (day 71), and whole blood (day 68). Only the brain biopsy sample was found to be positive for measles virus (MeV); >4,800,000 of 2 × 150 paired reads were assigned to the virus. These results were confirmed by specific MeV real-time reverse transcription PCR. 

**Figure 2:**
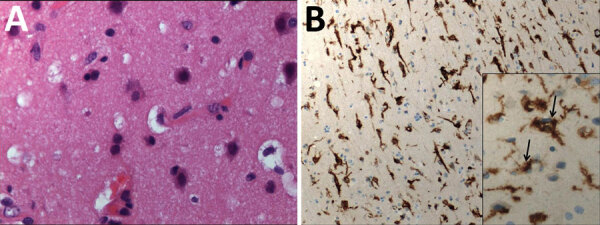
Histology and immunohistochemical staining of the cerebral cortex in a 28-year-old woman from Romania with untreated AIDS and measles inclusion-body encephalitis. A) Histology shows moderate increased cellular density and absence of nuclear inclusion bodies. Hematoxylin and eosin stain; original magnification ×100. B) Immunohistochemical staining microglial activation with high CD163 immunoreactivity Anti-human CD163 monoclonal antibody; original magnification ×100 and (inset) ×400.

The detected MeV sequences in the brain biopsy enabled the reconstruction of a nearly-complete genome assembly (99.5% with a median depth coverage >25,000; GenBank accession no. MN893225). BLAST analysis (https://blast.ncbi.nlm.nih.gov/Blast.cgi) showed this MeV exhibited 99.3% identity (15,762/15,876 nt) with the closest available fully sequenced B3 genotype (MVs/California.USA/05.14/[B3]; GenBank accession no. KY969477). These results excluded infection by a MeV vaccine strain and oriented toward a European lineage origin. However, it was not possible to determine the precise origin of the virus because a high number of MeV harboring an identical C-terminal hypervariable domain (450 nt) of the nucleoprotein N gene N-450, the only genetic data available on the World Health Organization Measles Nucleotide Surveillance database (http://www.who-measles.org) (*2*), were co-circulating in Europe at the time (data not shown) (*3**–**6*). In addition, we identified no previously reported mutations suspected for neurovirulence (*7**,**8*). 

We observed a mutational hotspot within the virus-encoded matrix protein (M). Of interest, the F1 5¢ end of the fusion protein (F) contained the most variable sites found along the genome. This hydrophobic F1 part is associated with hyperfusogenicity and neurodegenerative disorders (data not shown) (*7**,**8*).

Several characteristics supported a diagnosis of measles inclusion-body encephalitis (MIBE): an immunocompromised patient, undetectable MeV RNA with no intrathecal synthesis of MeV antibodies in CSF (MeV-specific IgG were detected in serum), and scarce inflammatory infiltrates on brain biopsy despite the absence of characteristic inclusions or multinucleated giant cells (*9*). Moreover, the retrospective clinical investigation revealed that the patient, who was not vaccinated against measles, had close contact with a sibling who was acutely ill with measles in April 2018 in Romania. Because of late MIBE diagnosis and despite supportive treatment, the patient’s neurologic status continued to deteriorate rapidly, and she died at day 109 with severe brain damage exemplified by pejorative MRI evolution (Figure 1), showing bilateral, symmetric, and diffuse distribution of lesions during days 59–94.

Ongoing measles resurgence may lead to an increase of measles-induced encephalitis cases with life-threatening outcomes. We report here a fatal case in a woman with AIDS who had an encephalitic syndrome with no initial clear etiologic diagnosis, retrospectively tagged as MIBE. The patient did not receive ribavirin therapy (*10*) for MeV infection because the first-line extensive diagnostic testing was unsuccessful. As a last resort, a SMg approach detected MeV in a brain biopsy, despite the known result that CSF MeV detection in MIBE is often negative (*9**–**11*). Of interest, the brain biopsy did not reveal histopathologic features consistent with MIBE; we observed no immunoreactive inclusions or multinucleated giant cells within glial cells or neurons (*10**,**11*). However, MIBE lesions are scanty and can be missed in a small biopsy sample. Unfortunately, there was no material available for electron microscopy, and an autopsy was not done. 

The encephalitic syndrome developed in this unvaccinated patient ≈6 months after a close contact with a documented measles case-patient; however, she did not report any rash or clinical symptoms of measles infection. It is noteworthy that MeV real-time reverse transcription PCR performed on the brain biopsy sample could have been sufficient to detect the virus. However, in this case, the advantage of SMg for the diagnosis of encephalitis is that, aside from pathogen identification, it was possible to generate full genome sequence for B3-genotype MeV. In conclusion, this case highlights the advantage to have a reliable pan-pathogen SMg tool to diagnose atypical encephalitis with no clear etiology on an early brain biopsy sampling.

## References

[1] Rodriguez C, Jary A, Hua C, Woerther P-L, Bosc R, Desroches M, et al.; Multidisciplinary Necrotizing Fasciitis Study Group. Pathogen identification by shotgun metagenomics of patients with necrotizing soft-tissue infections. Br J Dermatol. 2019. 10.1111/bjd.18611 31610037

[2] Rota PA, Brown K, Mankertz A, Santibanez S, Shulga S, Muller CP, et al. Global distribution of measles genotypes and measles molecular epidemiology. J Infect Dis. 2011;204(Suppl 1):S514–23. 10.1093/infdis/jir118 21666208

[3] Katoh K, Standley DM. MAFFT multiple sequence alignment software version 7: improvements in performance and usability. Mol Biol Evol. 2013;30:772–80. 10.1093/molbev/mst010 23329690 PMC3603318

[4] Drummond AJ, Rambaut A. BEAST: Bayesian evolutionary analysis by sampling trees. BMC Evol Biol. 2007;7:214. 10.1186/1471-2148-7-214 17996036 PMC2247476

[5] Drummond AJ, Ho SYW, Phillips MJ, Rambaut A. Relaxed phylogenetics and dating with confidence. PLoS Biol. 2006;4:e88. 10.1371/journal.pbio.0040088 16683862 PMC1395354

[6] Darriba D, Taboada GL, Doallo R, Posada D. jModelTest 2: more models, new heuristics and parallel computing. Nat Methods. 2012;9:772. 10.1038/nmeth.2109 22847109 PMC4594756

[7] Hashiguchi T, Fukuda Y, Matsuoka R, Kuroda D, Kubota M, Shirogane Y, et al. Structures of the prefusion form of measles virus fusion protein in complex with inhibitors. Proc Natl Acad Sci USA. 2018;115:2496–501. 10.1073/pnas.1718957115 PMC5877970 29463726

[8] Plattet P, Alves L, Herren M, Aguilar HC. Measles virus fusion protein: structure, function and inhibition. Viruses. 2016;8:112. 10.3390/v8040112 27110811 PMC4848605

[9] Fisher DL, Defres S, Solomon T. Measles-induced encephalitis. QJM. 2015;108:177–82. 10.1093/qjmed/hcu113 24865261

[10] Baldolli A, Dargère S, Cardineau E, Vabret A, Dina J, de La Blanchardière A, et al. Measles inclusion-body encephalitis (MIBE) in a immunocompromised patient. J Clin Virol. 2016;81:43–6. 10.1016/j.jcv.2016.05.016 27315036

[11] Bitnun A, Shannon P, Durward A, Rota PA, Bellini WJ, Graham C, et al. Measles inclusion-body encephalitis caused by the vaccine strain of measles virus. Clin Infect Dis. 1999;29:855–61. 10.1086/520449 10589903

